# Intramyocardial bone marrow cell injection does not lead to functional improvement in patients with chronic ischaemic heart failure without considerable ischaemia

**DOI:** 10.1007/s12471-018-1213-2

**Published:** 2018-12-19

**Authors:** I. Mann, C. C. S. Tseng, S. F. Rodrigo, S. Koudstaal, J. van Ramshorst, S. L. Beeres, P. Dibbets-Schneider, L. F. de Geus-Oei, H. J. Lamb, R. Wolterbeek, J. J. Zwaginga, W. E. Fibbe, K. Westinga, J. J. Bax, P. A. Doevendans, M. J. Schalij, S. A. J. Chamuleau, D. E. Atsma

**Affiliations:** 1 Department of Cardiology, Leiden University Medical Center, Leiden, The Netherlands; 2 Department of Cardiology, University Medical Center Utrecht, Utrecht, The Netherlands; 3 Department of Radiology, Leiden University Medical Center, Leiden, The Netherlands; 4 MIRA Institute for Biomedical Technology and Technical Medicine, University of Twente, Enschede, The Netherlands; 5 Department of Medical Statistics and Bioinformatics, Leiden University Medical Center, Leiden, The Netherlands; 6 Department of Hematology, Leiden University Medical Center, Leiden, The Netherlands; 7 Department of Cell Therapy Facility, University Medical Center Utrecht, Utrecht, The Netherlands

**Keywords:** Chronic heart failure, Ischaemia, Bone marrow cells

## Abstract

**Background:**

It has been suggested that bone marrow cell injection may have beneficial effects in patients with chronic ischaemic heart disease. However, previous trials have led to discrepant results of cell-based therapy in patients with chronic heart failure. The aim of this study was to evaluate the efficacy of intramyocardial injection of mononuclear bone marrow cells in patients with chronic ischaemic heart failure with limited stress-inducible myocardial ischaemia.

**Methods and results:**

This multicentre, randomised, placebo-controlled trial included 39 patients with no-option chronic ischaemic heart failure with a follow-up of 12 months. A total of 19 patients were randomised to autologous intramyocardial bone marrow cell injection (cell group) and 20 patients received a placebo injection (placebo group). The primary endpoint was the group difference in change of left ventricular ejection fraction, as determined by single-photon emission tomography. On follow-up at 3 and 12 months, change of left ventricular ejection fraction in the cell group was comparable with change in the placebo group (*P* = 0.47 and *P* = 0.08, respectively). Also secondary endpoints, including left ventricle volumes, myocardial perfusion, functional and clinical parameters did not significantly change in the cell group as compared to placebo. Neither improvement was demonstrated in a subgroup of patients with stress-inducible ischaemia (*P* = 0.54 at 3‑month and *P* = 0.15 at 12-month follow-up).

**Conclusion:**

Intramyocardial bone marrow cell injection does not improve cardiac function, nor functional and clinical parameters in patients with severe chronic ischaemic heart failure with limited stress-inducible ischaemia.

Clinical Trial Registration: NTR2516

**Electronic supplementary material:**

The online version of this article (10.1007/s12471-018-1213-2) contains supplementary material, which is available to authorized users.

## Introduction

In patients with ischaemic heart disease, myocardial damage can lead to remodelling of the left ventricle and progress towards end-stage heart failure (HF) [1]. Despite major advances in medical and surgical options for the management of ischaemic heart disease no definite cure is available for HF. Moreover, severe chronic HF has a poor prognosis with a one-year mortality rate of 50% in patients with severe HF symptoms (New York Heart Association [NYHA] score 4) [2, 3]. Many chronic HF patients remain symptomatic, causing a large burden on day-to-day activities, as well as on health care usage and costs. Therefore, there is a need for new therapeutic strategies to treat chronic ischaemic HF.

Bone marrow cells have emerged as a potential therapy since they were hypothesised to stimulate angiogenesis by the release of growth factors and/or by direct incorporation of cells into new capillaries [4–6]. Extrapolated from this hypothesis, bone marrow cell treatment might benefit ischaemic myocardium and lead to improvement in cardiac function and symptoms. The first clinical trials with bone marrow cells were performed in patients after an acute myocardial infarction [7, 8] and showed contradictory results with regard to beneficial effects. Bone marrow cells have also been evaluated in patients with chronic ischaemia and refractory angina pectoris with optimised therapy and without long-term treatment options (‘no-option’) [9–11]. These latter trials demonstrated that intramyocardial injections with bone marrow cells are safe and result in improvement of cardiac function, myocardial perfusion and anginal symptoms [9–11]. Intramyocardial bone marrow cell injection in patients with chronic HF has been demonstrated to be safe and feasible [12–14]. However, since most of these trials included patients with complaints of angina pectoris and/or (objectified) ischaemia, the efficacy in patients without (chronic) stress-inducible ischaemia is unclear [14–17]. Up to now, there have been no clinical studies that evaluated whether the presence or absence of stress-inducible ischaemia influences the outcome of bone marrow cell treatment in patients with ischaemic HF. In patients with dilated cardiomyopathy, the majority of studies show a significant increase in left ventricular function after cell treatment, although no solid evidence exists [18].

As there is still a need for novel therapies in no-option HF patients, the aim of the current randomised, double-blind, placebo-controlled multicentre study is to evaluate the efficacy of intramyocardial bone marrow cell injection in patients with chronic ischaemic HF regardless of the presence of stress-inducible ischaemia. Furthermore, this study aimed to investigate whether the presence of stress-inducible myocardial ischaemia influences the outcome of bone marrow cell treatment in these patients.

## Methods

### Study overview

The present study is a phase 2, randomised, double-blind, placebo-controlled multicentre trial. The participating centres were the Leiden University Medical Center (LUMC) and the University Medical Center of Utrecht (UMCU). The LUMC has been the coordinating centre that provided trial management and data analysis. The study protocol was in accordance with the declaration of Helsinki and complied with the Guideline for Good Clinical Practice (CMPP/ICH/135/95—17th July 1996). The protocol was approved by the institutional ethical committees of both research centres and the Dutch Central Committee on Research Involving Human Subjects (CCMO). Overall safety examination was performed by an independent data monitoring safety board (DSMB), as well as by independent institutional safety review boards of each clinical centre. The study has been registered at the Dutch trial registry (www.trialregister.nl, no. NTR2516).

### Population

**Fig. 1:**
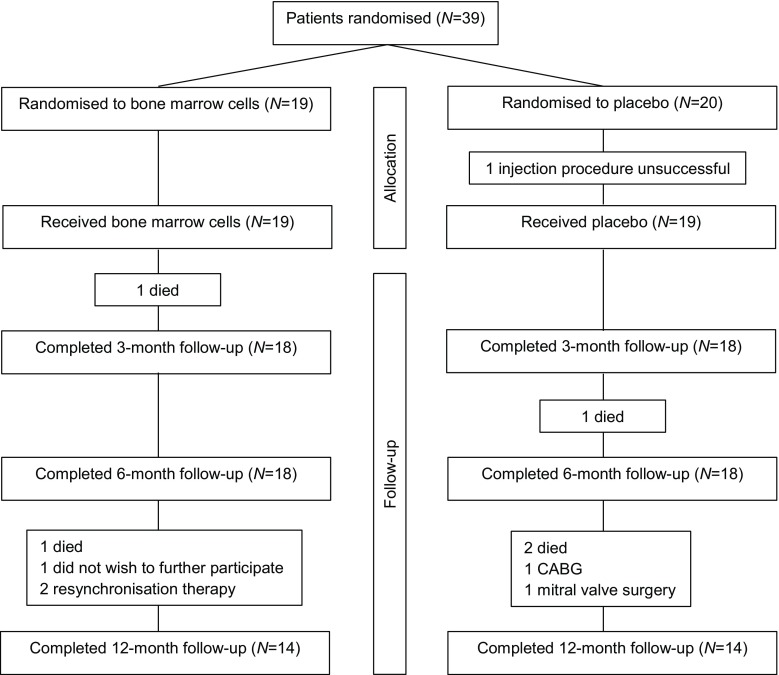
Patient flow diagram. (*CABG* coronary artery bypass grafting)

The study population consisted of patients with coronary artery disease and chronic HF (NYHA class 2, 3 or 4) despite optimised medical therapy, and were recruited by the 2 participating centres. Full inclusion and exclusion criteria are provided in Tab. 1. A 1:1 randomisation was executed by a statistician of the LUMC (Fig. 1). The randomisation was stratified by presence of stress-inducible ischaemia, to assure a similar amount of patients with and without stress-inducible ischaemia in both treatment and placebo group, and by clinical centre, to equally assign patients who underwent cell-based therapy versus placebo to both hospitals.

**Table 1:** Inclusion/exclusion criteria

*Inclusion criteria*
– Ischaemic heart failure NYHA class 2, 3 or 4 despite optimal pharmacological and non-pharmacological therapy – No candidate for (repeat) surgery (revascularisation, valve repair or ventricular reconstruction) – No candidate for (repeat) percutaneous revascularisation – Receiving resynchronisation therapy when indicated – Male or female >18 years – Life expectancy more than 6 months – Able to perform an exercise test prior to therapy – Able and willing to undergo all the tests used in this protocol including the traveling involved – Written informed consent
*Exclusion criteria*
– Evidence of cancer in the last 5 year (except low-grade and fully resolved non-melanoma skin malignancy) – Concurrent participation in a study using an experimental drug or an experimental procedure within 2 months before randomisation – Other severe concurrent illnesses (including active infection, aortic stenosis defined as aortic valve area below 1.0 cm^2^, severe renal insufficiency defined as a GFR <30 ml/min/1.73 m^2^) – Bleeding diathesis, HIV infection or pregnancy – Any other condition that, in the opinion of the investigator, could pose a significant threat to the subject if the investigational therapy is initiated – Inability to undergo cardiac catheterisation or nuclear imaging – Inability to follow the protocol and comply with follow-up requirements – Candidate for surgery (revascularisation, valve repair or ventricular reconstruction), resynchronisation therapy or percutaneous revascularisation

*NYHA* New York Heart Association, *HIV* human immunodeficiency virus, *GFR* glomerular filtration rate

### Study protocol

At baseline, a complete medical history was obtained and laboratory tests including blood count, electrolytes, cardiac markers, hepatic and renal function, and infectious disease serology were performed. Further baseline evaluation consisted of stress-rest Tc-99m tetrofosmin gated single-photon emission tomography (SPECT), fluorodeoxyglucose (FDG) SPECT, metaiodobenzylguanidine (MIGB) imaging, NYHA classification, quality of life assessment using the Dutch translation of the Minnesota Living with Heart Failure Questionnaire (MLHF), and a bicycle exercise test with volume oxygen maximum (VO2 max) measurement. Next, patients were hospitalised for bone marrow aspiration and the intramyocardial injection procedure. To evaluate peri-procedural and short-term safety of bone marrow cell treatment, patients were admitted to the Coronary Care Unit immediately after treatment. Monitoring of vital signs, heart rhythm and biochemical cardiac markers were performed during 2 days after treatment. Before discharge, echocardiography was performed to evaluate the presence of pericardial effusion. A follow-up SPECT was repeated at 3 and 12 months. NYHA class was reassessed on follow-up at 3, 6 and 12 months, and MLHF and bicycle test were repeated at 3 and 6 months. We evaluated long-term safety during outpatient visits at 1.5, 3, 6 and 12 months after treatment to ensure the collection of all adverse events, including all-cause death, cardiovascular death, hospitalisation for worsening HF, rhythm disorders and myocardial infarction. A 24-hour Holter recording was obtained in patients without implantable cardioverter defibrillator (ICD) at 1.5 and 6 months to monitor the occurrence of arrhythmias.

### Bone marrow aspiration and cell processing

On the day of the injection procedure, ≥80 ml of bone marrow was aspirated from the posterior iliac crest under local anaesthesia. During the procedure patients were under continuous ECG and oxygen saturation registration. Bone marrow was collected in flasks containing Hanks balance salt solution and heparin. Mononuclear bone marrow cells were isolated by Ficoll density gradient centrifugation according to strict GMP conditions following Standard Operating Procedures of the Stem Cell Laboratory at the LUMC (CST-WV-2014, CST-WR 2014, CST0PF-2000.BM/MNC and CST-Do-2000). Bone marrow cells were suspended at a concentration of 40 × 10^6^ cells/ml in a solution of 0.9% sodium chloride, 0.5% human albumin and 11.3 IU/ml heparin [9]. The final cell product contained 100 × 10^6^ cells. The bone marrow cell population was checked for clots, stained for the presence of bacteria and analysed by fluorescence-activated cell sorting for the presence and percentage of CD14-positive, CD34-positive and CD45-positive cells [9].

A blinded syringe with either bone marrow solution or placebo solution, which contained a similar sodium chloride/human albumin solution without bone marrow-derived cells, was delivered at the catheterisation laboratory. Patient’s allocation was only known to the stem cell laboratory assistant.

### Electromechanical mapping and cell injection

During cell preparation, biplane left ventricular angiography was performed. Based on ventricular size, the mapping catheter curve (D or F) was selected. Via femoral artery access and a retrograde aortic approach, the catheter was inserted into the left ventricle. A non-fluoroscopic electromechanical map of the left ventricle was constructed using the NOGA system (NOGAStar catheter, Biosense-Webster, Waterloo, Belgium) [19]. The electromechanical map was used to guide the injection catheter with a 27-gauge retractable needle (MyoStar catheter Biosense-Webster) to the target region, which is the area of stress-inducible ischaemia on SPECT for the patients with ischaemia [19], and to the peri-infarction zone for patients without ischaemia [20]. Subsequently, 8–12 injections of approximately 0.2–0.3 ml each were delivered.

### SPECT

Gated SPECT imaging was performed with a triple-head SPECT camera system (GCA 9300/HG, Toshiba Corp., Tokyo, Japan). A 2-day stress-rest protocol was used for Tc-99m tetrofosmin SPECT imaging. Since the infusion of adenosine should be continued for 2–3 minutes after injection of the radiopharmaceutical, adenosine was given as a continuous infusion (0.14 mg/kg/min) for 6 minutes with injection of Tc-99m tetrofosmin (500 MBq) at 3 minutes after the start of the adenosine infusion. Stress imaging was performed 45 minutes after injection. On the second day, rest images were obtained 1 hour after injection of Tc-99m tetrofosmin (500 MBq). To obtain left ventricular ejection fraction (LVEF) and left ventricular volumes, rest imaging studies were acquired using ECG gating [9]. We carried out a quantitative assessment of left ventricular end-systolic and end-diastolic volumes and LVEF at rest and stress using the 4DM software (Corridor 4DM, INVIA, Ann Arbor, University of Michigan Medical Center) [21]. To analyse myocardial perfusion, the gated SPECT images were divided in 17 segments and we categorised segmental tracer uptake, at rest and stress imaging, on a 4 point scale: 1 = tracer activity >75%; 2 = tracer activity 50–75%; 3 = tracer activity 25–49%; 4 = tracer activity <25%. When segmental tracer uptake during stress was >75% of maximum tracer uptake, perfusion was considered to be normal [22]. The summed stress score was calculated by summation of the segmental scores at stress and summed rest score was calculated by summation of the segmental scores at rest. The summed differences score was calculated by summation of the differences in stress and rest segmental scores and reflects the extent of stress-inducible ischaemia. A summed difference score of 1 was defined as presence of stress-inducible ischaemia.

### FDG SPECT

FDG imaging was performed to assess myocardial viability [23]. The plasma glucose level was assessed and regulated by an oral dose of 500 mg acipimox and 45 minutes later a low-fat carbohydrate-rich meal [23]. FDG (185 MBq) was injected two hours thereafter, tracer uptake imaging was performed after 45 minutes of rest using the dual-head Toshiba, GCA 7200A/DI ultra high-energy collimator. Myocardial tracer uptake was scored on a 17 segment model and scored on a 4-point scale: 1 = normal tracer uptake (activity >75%); 2 = mildly reduced tracer uptake (activity 50–75%); 3 = moderately reduced tracer uptake (activity 25–49%); 4 = severely reduced tracer uptake (activity <25%). Segments with >75% of maximum tracer uptake were considered as viable (normal) and segments with tracer uptake <75% were considered to contain some extent of scar. An increase in segmental uptake by 1 point was considered an improvement [23].

### MIBG imaging

^123^I-MIBG imaging was performed to assess myocardial (sympathetic) innervation. Patients were pre-treated with sodium iodide or potassium iodide 1 hour prior to injection to block uptake of free ^123^Iodine by the thyroid gland. 185 MBq of ^123^I-MIBG was injected and ^123^I-MIBG planar imaging was performed in the supine position after 15 minutes and 4 hours. The heart-to-mediastinum ratio was calculated for the late planar images [24].

### Exercise test

Exercise capacity was assessed by a bicycle exercise test. Patients performed a symptom-limited bicycle exercise test with a 20W starting load and an increment of 10 W/min, including VO2max measurement. Test endpoints were angina pectoris, physical exhaustion, dyspnoea and significant decrease in systolic blood pressure (>10 mm Hg), as measured every 2 minutes [9].

### Statistical analysis

This trial was originally powered at 80% (*α* < 0.05) to demonstrate a minimum of 4.1% ± 5.4% difference in LVEF at rest as assessed by SPECT between subjects randomised to autologous bone marrow cell therapy (28 patients) compared with placebo-controlled subjects (28 patients) at 3‑month follow-up. To account for a 15% dropout rate, we had to include 32 patients in each group. Due to slow inclusion, however, we decided to terminate inclusion 4 years after the first included patient, regardless of the number of patients at that time point.

Primary endpoint was defined as the difference in change of LVEF, determined by SPECT, between baseline and follow-up. A prespecified composite endpoint consisted of LVEF, summed stress score (SPECT), NYHA class, MLHF score, exercise capacity, VO2 max, 6‑minute walking distance, viability (FDG SPECT) and heart-to-mediastinum ratio (MIBG). Improvement per patient was quantified on a scale ranging from −9 (deterioration on every test) to +9 (improvement on every test), baseline compared to 3‑month follow-up. A subanalysis was performed to compare the effect of cell-based therapy in patients with and without stress-inducible ischaemia.

Continuous data were compared using student’s t‑test and presented as mean ± SD and categorical variables were analysed using Chi-squared test and presented as numbers and percentage. To analyse changes during multiple follow-up time points, we used a repeated measure model (mixed model) with time as repeated measurement (covariance type; Toeplitz heterogeneous). Taken into account similarity of both groups at baseline, the interaction between treatment allocation (factor) and follow-up time points (covariate) is used to analyse the treatment effect (between-group differences at each follow-up time point). Data is presented as estimated difference with 95% confidence interval (CI) between baseline and follow-up time points within groups as well as between groups. Data were analysed following the intention-to-treat principle. A *P*-value <0.05 is considered statistically significant.

### Safety monitoring

Serious adverse events (SAEs) and suspected unexpected serious adverse reactions (SUSARs) were reported to the CCMO, as well as to the DSMB. The DSMB evaluated whether adverse events in the patients included in the study were related to any of the diagnostic or therapeutic procedures used in the protocol. Furthermore, an annual safety report was submitted to the CCMO and DSMB. This provided an overview of all SUSARs and SAEs, accompanied by a brief report, highlighting the main points of concern.

## Results

Between April 2010 and June 2014, patients were enrolled in the study. It proved not possible to include the intended number of patients fully complying with the inclusion criteria within these 4 years. A total of 39 patients, approximately 50% of all screened patients, were enrolled (Fig. 1). The majority of screen failures were attributed to a lack of consent due to the placebo risk (20%), NYHA class I (20%) and other treatment options (15%). After randomisation, 19 patients were treated with bone marrow mononuclear cells and 20 patients with a placebo suspension. Baseline characteristics of enrolled subjects are presented in Tab. 2. Patients of both groups were comparable regarding age, medical history and clinical status. A total of 21 patients were treated in the LUMC and 18 patients in the UMCU.

**Table 2:** Baseline characteristics

	Cell injection (*n* = 19)	Placebo (*n* = 20)	*P*-value
*Age, mean* *±* *SD, y*	65 ± 7	65 ± 8	0.61
*Men*	19 (100%)	18 (90%)	0.49
*Cardiovascular risk factors*			
Current smoking	2 (11%)	3 (15%)	1.00
History of smoking	16 (84%)	14 (70%)	0.45
Hypertension	12 (63%)	9 (45%)	0.26
Diabetes	5 (26%)	5 (25%)	1.00
Dyslipidaemia	14 (74%)	14 (70%)	0.80
Family history of CAD	14 (74%)	9 (45%)	0.07
BMI, mean ± SD, kg/m^2^	29 ± 5	29 ± 5	0.44
*Medication*			
Nitrates	9 (47%)	11 (55%)	0.63
Beta-blockers	17 (89%)	16 (80%)	0.66
Calcium channel blockers	3 (16%)	7 (35%)	0.27
Statins	17 (89%)	15 (75%)	0.41
ACE inhibitors	12 (63%)	12 (60%)	0.84
ATII antagonist	9 (47%)	7 (35%)	0.43
Clopidogrel	2 (11%)	6 (30%)	0.24
Aspirin	3 (16%)	9 (45%)	0.05
OAC	16 (84%)	11 (55%)	0.05
Diuretics	15 (79%)	15 (75%)	1.00
*Medical history*			
ICD	15 (79%)	14 (70%)	0.72
PM	1 (5%)	0 (0%)	0.49
Biventricular pacing	5 (26%)	6 (30%)	0.80
Prior MI	18 (95%)	20 (100%)	0.49
Prior CABG	12 (63%)	12 (60%)	0.84
Prior PCI	11 (58%)	14 (70%)	0.43
Prior CVA/TIA	2 (11%)	3 (15%)	1.00

*IDDM* insulin-dependent diabetes mellitus, *NIDDM* non-insulin-dependent diabetes mellitus, *CAD* coronary artery disease, *BMI* body mass index, *ACE* angiotensin-converting enzyme, *AT* angiotensin, *OAC* oral anticoagulants, *ICD* internal cardiac defibrillator, *PM* pacemaker, *MI* myocardial infarction, *CABG* coronary artery bypass grafting, *PCI* percutaneous coronary intervention, *CVA/TIA* cerebrovascular accident/transient ischaemic attack

Safety data were collected up to 12 months in 34 patients or until death in 5 patients. Functional and clinical data were collected up to 12 months in 28 patients, until death in 5 patients, until significant medical intervention in 4 patients, or in case of 1 patient, up to the moment the patient wished to no longer participate, which was mainly determined by major comorbidities (pulmonary emphysema). In 1 patient the intramyocardial injection procedure was unsuccessful due to the inability to reach the heart with the NOGA catheter as a result from severe bilateral femoral artery stenosis. Therefore, no functional or clinical follow-up data was collected in this patient.

### Safety data

During 12-month follow-up, 2 cell-treated patients and 3 placebo-treated patients died. One of the cell-treated patients died 2.5 months after the injections. The other deceased cell-treated patient requested euthanasia at 7.5 months after the procedure because of incurable suffering from chronic pain that was not related to his cardiac disease. Of the placebo-treated patients, 1 patient died from complication of acute myocardial ischaemia 7 months after the procedure, 1 patient died following an out-of-hospital cardiac arrest 6.5 months after the procedure, and the other patient died from cardiorespiratory insufficiency due to terminal HF 3.5 months after the procedure.

In 2 cell-treated and 2 placebo-treated patients a serious adverse event resulted in medical intervention and exclusion of further follow-up. Both cell-treated patients underwent cardiac resynchronisation therapy (CRT). In 1 of these patients, at 6‑month follow-up after a high-grade atrioventricular block and pacemaker dependency, a CRT was indicated and in the other patient at 10-month follow-up, a CRT was indicated because of progressive intraventricular conduction delay. One placebo-treated patient had a non-ST-elevation myocardial infarction 9 months after the procedure and underwent coronary artery bypass grafting. The other placebo-treated patient was admitted at 7‑month follow-up because of decompensated HF and underwent minimal invasive mitral valve repair.

Other SAEs included a monomorphic ventricular tachycardia in a cell-treated patient at 1.5 months, which was stabilised with amiodarone, and a near collapse due to a non-sustained ventricular tachycardia in a placebo-treated patient at 6.5 months. All SAEs are summarised in supplementary Tab. 1.

### Procedural data

Mean procedural time was 112 ± 40 minutes in the bone marrow cell group and 110 ± 44 minutes in the placebo group (*P* = 0.87). In both groups, 19 patients received intramyocardial injections. Patients in the bone marrow cell group received 9.5 ± 0.8 injections and patients in the placebo group received 9.1 ± 1.3 injections (*P* = 0.29). All patients from the cell group received 100X10^6^ cells, with a CD34+ fraction of 1.6% ± 0.6%.

### LVEF and volumes

At baseline all 39 patients underwent SPECT. At 3 months paired SPECT studies were available in 18 patients from both treatment groups due to previously described deaths (*N* = 2) and unsuccessful injection procedure (*N* = 1). At 12-month follow-up, SPECT scans of 14 cell-treated patients and 13 placebo-treated patients were available (missing scans due to previously described reasons, 1 scan unavailable due to logistics reasons).

**Fig. 2:**
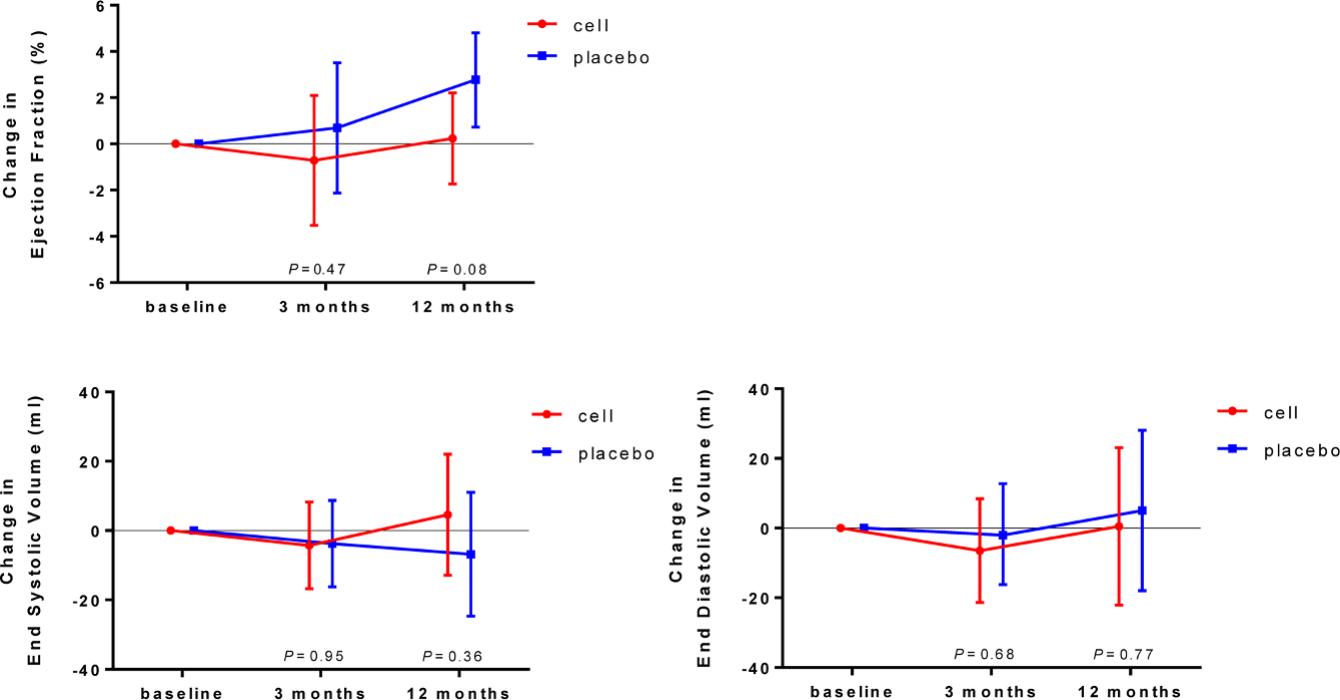
Change in left ventricular ejection fraction and volumes. Mean estimated changes at 3‑month and 12-month follow-up in left ventricular ejection fraction at rest (above) and end-systolic and end-diastolic volumes. Bars represent 95% confidence intervals. The treatment effect (difference in changes between bone marrow cell injection group and placebo group) is not significant

In the cell-treated group no improvement in LVEF at rest, assessed by SPECT, was detected (*P* = 0.61 at 3‑month and *P* = 0.81 at 12-month follow-up). Also in the placebo group, at 3‑month follow-up LVEF at rest did not change (*P* = 0.62). In the placebo group at 12-month follow-up, there was a significant increase in LVEF (*P* = 0.01). However, we did not detect any significant differences (LVEF change in percentage points from baseline) between both groups at 3‑month and 12-month follow-up (respectively, *P* = 0.47 and *P* = 0.08). We also did not observe any differences between groups in LVEF at stress, left ventricular end-systolic volume and left ventricular end-diastolic volume at 3‑month and 12-month follow-up, as shown in Tab. 3 and Fig. 2.

**Table 3:** Left ventricular function and perfusion

*Bone marrow cell group*											
	*Baseline (N* *=* *19)*	*3-month follow-up* *(N* *=* *18)*					*12-month follow-up* *(N* *=* *14)*				
	Absolute value (mean ± SD)	Absolute change (mean ± SD)	Estimated difference (mean)	95% CI		*P*-value	Absolute change (mean ± SD)	Estimated difference (mean)	95% CI		*P*-value
				Lower	Upper				Lower	Upper	
EFr (%)	32.6 ± 12.3	−1.0 ± 7.0	−0.7	−3.5	2.1	0.61	0.6 ± 4.3	0.2	−1.7	2.2	0.81
EFs (%)	32.2 ± 12.2	−1.9 ± 7.6	−1.4	−4.2	1.4	0.32	1.0 ± 7.5	1.2	−2.0	4.5	0.45
ESV (ml)	220 ± 126	−3.7 ± 23.4	−4.3	−16.8	8.2	0.49	2.9 ± 30.6	4.5	−12.9	22.0	0.59
EDV (ml)	309 ± 131	−6.7 ± 26.5	−6.5	−21.4	8.4	0.39	3.7 ± 36.6	0.5	−22.1	23.1	0.96
SSS	46.4 ± 7.4	−0.4 ± 1.5	−0.5	−1.2	0.3	0.20	0.2 ± 2.5	−0.1	−1.3	1.0	0.86
SRS	45.1 ± 7.6	−0.1 ± 1.9	−0.1	−1.1	0.9	0.85	0.4 ± 2.4	−0.1	−1.4	1.3	0.87
SDS	1.3 ± 2.2	−0.4 ± 1.0	−0.6	−1.2	−0.01	0.05	−0.1 ± 1.8	−0.3	−1.1	0.5	0.48
*Placebo group*											
	*Baseline (N* *=* *20)*	*3-month follow-up* *(N* *=* *18)*					*12-month follow-up* *(N* *=* *13)*				
	Absolute value (mean ± SD)	Absolute change (mean ± SD)	Estimated difference (mean)	95% CI		*P*-value	Absolute change (mean ± SD)	Estimated difference (mean)	95% CI		*P*-value
				Lower	Upper				Lower	Upper	
EFr (%)	29.0 ± 9.7	0.8 ± 4.8	0.7	−2.1	3.5	0.62	2.6 ± 2.7	2.8	0.7	4.8	0.01
EFs (%)	29.0 ± 9.0	−0.3 ± 4.6	−0.6	−3.3	2.2	0.68	2.1 ± 5.1	0.9	−2.3	4.2	0.56
ESV (ml)	221 ± 105	−3.7 ± 34.0	−3.8	−16.2	8.7	0.55	−9.7 ± 31.5	−6.8	−24.7	11.0	0.44
EDV (ml)	299 ± 110	−2.2 ± 43.4	−2.0	−16.9	12.8	0.78	−2.2 ± 38.2	5.0	−18.0	28.1	0.65
SSS	46.8 ± 4.5	−0.9 ± 1.9	−0.8	−1.6	−0.1	0.03	−0.5 ± 2.2	−0.7	−1.9	0.5	0.23
SRS	44.0 ± 5.1	0.3 ± 1.0	0.4	−0.3	1.0	0.25	0.4 ± 1.5	0.3	−0.7	1.32	0.56
SDS	2.7 ± 3.1	−1.2 ± 1.7	−1.1	−1.7	−0.45	<0.01	−0.8 ± 1.9	−0.9	−1.7	−0.1	0.04
*Group difference (treatment effect)*											
	*Baseline*	*3-month follow-up*					*12-month follow-up*				
	Absolute difference (mean)	Absolute difference in change (mean)	Estimated difference (mean)	95% CI		*P-*value	Absolute difference in change (mean)	Estimated difference (mean)	95% CI		*P-*value
				Lower	Upper				Lower	Upper	
EFr (%)	3.7	−1.8	−1.4	−5.4	2.5	0.47	−2.0	−2.5	−5.4	0.3	0.08
EFs (%)	3.2	−1.6	−0.8	−4.6	3.0	0.67	−1.1	0.3	−4.1	4.7	0.89
ESV (ml)	−0.7	0.0	−0.5	18.1	17.0	0.44	12.5	11.4	−13.5	36.3	0.36
EDV (ml)	10.8	−4.6	−4.4	−25.5	16.6	0.68	5.9	−4.5	−36.8	27.7	0.77
SSS	−0.3	0.4	0.4	−0.7	1.4	0.48	0.7	0.6	−1.0	2.2	0.45
SRS	1.1	−0.4	−0.4	−1.3	0.4	0.33	0.0	−0.4	−1.8	1.0	0.59
SDS	−1.4	0.8	0.4	−0.3	1.2	0.25	0.7	0.6	−0.4	1.7	0.24

*CI* confidence interval, *EFr* ejection fraction at rest, *EFs* ejection fraction at stress, *ESV* end-systolic volume, *EDV* end-diastolic volume, *SSS* summed stress score, *SRS* summed rest score, *SDS* summed differences score

### Myocardial perfusion and stress-inducible ischaemia

Summed stress score did not significantly change in the cell group (*P* = 0.20 at 3 months and *P* = 0.86 at 12 months follow-up). In the placebo group at 3‑month follow-up, there was a small improvement in summed stress score (*P* = 0.03). However this improvement did not sustain up to 12 months (*P* = 0.21) and was not significantly different compared with the cell group (*P* = 0.48 at 3‑month and *P* = 0.45 at 12-month follow-up).

There were no changes in summed rest score in the cell group (*P* = 0.89 at 3‑month and *P* = 0.87 at 12-month follow-up) or in the placebo group (*P* = 0.25 at 3‑month and *P* = 0.56 at 12-month follow-up). This was comparable between both groups (*P* = 0.33 at 3‑month and *P* = 0.59 at 12-month follow-up).

There was a small but significant improvement in summed difference score in the cell group at 3‑month follow-up (*P* = 0.05), which did not sustain up to 12-month follow-up (*P* = 0.48). In the placebo group there was also an improvement at 3 months, which sustained up to 12-month follow-up (respectively, *P* < 0.01 and *P* = 0.03). Importantly, no significant differences between both groups were detected (respectively, *P* = 0.25 and *P* = 0.24). Data are shown in Tab. 3.

### Myocardial viability

Baseline F18-FDG SPECT images were available for all cell-treated and 17 placebo-treated-patients and for 16 of both cell-treated and placebo-treated patients at 3‑month follow-up. In the cell group at 3‑month follow-up, there was a small but significant improvement in viability score (−1.2 [95% CI −2.4–−0.1]; *P* = 0.05), which was not seen after placebo treatment (−0.2 [95% CI −1.4–1.0]; *P* = 0.72). However, no significant treatment effect was observed (group difference −1.0 [95% CI −2.6–0.6]; *P* = 0.22).

### Myocardial innervation

All patients underwent baseline 123-I-MIBG SPECT imaging and at 3‑month follow-up, 18 scans of both groups were available for analysis. At 3‑month follow-up, late heart-to-mediastinum ratio remained unchanged in both cell group and placebo group (estimated mean difference −0.01 [95% CI −0.1–0.1]; *P* = 0.77 and −0.01 [95% CI −0.1–0.1]; *P* = 0.75 respectively, group difference 0.001 [95% CI −0.1–0.1]; *P* = 0.99).

### Clinical outcome

Clinical status, assessed by NYHA score, did not change in both treatment groups on follow-up at 3, 6 and 12 months (group difference *P* = 0.73, *P* = 0.13 and *P* = 0.52, respectively).

**Fig. 3:**
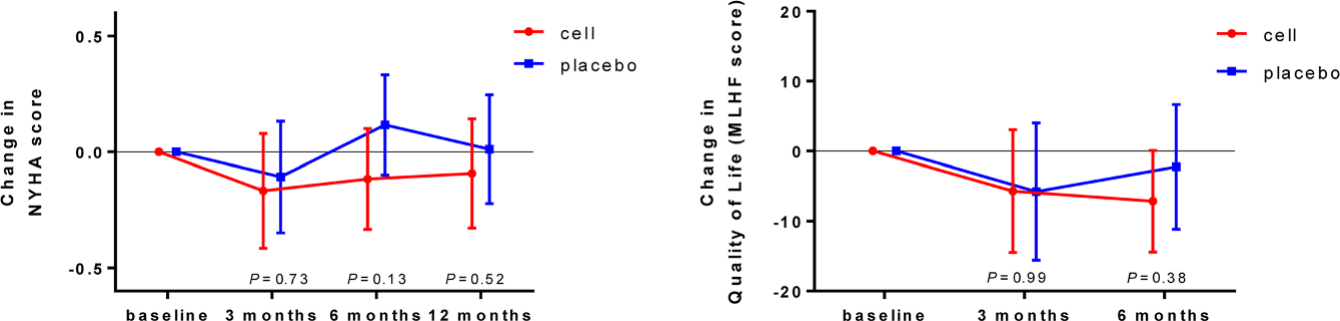
Change in NYHA score and quality of life. Mean estimated changes on follow-up at 3, 6 and 12 months in NYHA score and on follow-up at 3 and 6 months in MLHF score. Bars represent 95% confidence intervals. The treatment effect (difference in changes between bone marrow cell injection group and placebo group) is not significant, as shown by *P*-values. (*NYHA* New York Heart Association, *MLHF* Minnesota living with heart failure questionnaire)

Improvement in quality of life MLHF score after 3 months in the cell group was not significant (estimated mean difference −5.7 [95% CI −14.5–3.0]; *P* = 0.19). However, at 6 months there was a trend to improvement (estimated mean difference −7.2 [95% CI −14.4–0.01]; *P* = 0.05). In the placebo group, improvement in quality of life score was not significant (estimated mean difference at 3 months −5.8 [95% CI −15.6–4.0]; *P* = 0.24 and at 6 months −2.2 [95% CI −11.1–6.7]; *P* = 0.61). Nevertheless, between-group differences were not significant (*P* = 0.99 at 3‑month and *P* = 0.38 at 6‑month follow-up). Data are presented in supplementary Tab. 2 and Fig. 3.

### Exercise capacity

There were no significant changes in exercise capacity assessed by bicycle test, after cell or placebo treatment. Both groups were comparable at 3 and 6 months (*P* = 0.51 and *P* = 0.36, respectively). Similarly, we did not find any significant changes in VO2max during the bicycle test (group differences at 3 months *P* = 0.82 and at 6 months *P* = 0.42). Data are presented in supplementary Tab. 2.

### Ischaemia

Prespecified subgroup analysis examined the efficacy of cell-based therapy in patients with stress-inducible ischaemia. At baseline, in the cell group 8 patient had ischaemia (mean summed difference score 3.1 ± 2.4) and in the placebo group 13 patients had ischaemia (mean summed difference score 4.2 ± 3.0) Therefore, 21 patients were included in the analysis. Baseline LVEF was 29.6 ± 11.5% in the cell group and 29.7 ± 11.5 in the placebo group. Estimated mean difference in the cell group was −0.3% (95% CI −5.0–4.4%); *P* = 0.91 at 3‑month follow-up and −0.5% (95% CI −2.9–1.8%); *P* = 0.64 at 12-month follow-up. In the placebo group estimated mean differences were 1.5% (95% CI −2.1–5.1%); *P* = 0.39 at 3‑month follow-up and 1.7% (95% CI −0.5–3.9%); *P* = 0.12 at 12-month follow-up. Between-group difference at 3 months was −1.8% (95% CI −7.7–4.1%); *P* = 0.54 at 3‑month follow-up and −2.2% (95% CI −5.4–0.9%); *P* = 0.15 at 12-month follow-up. Also, no significant changes were observed between ischaemic cell-treated and ischaemic placebo-treated patients in secondary endpoints (data not shown).

## Discussion

This reported multi-centre, double blind, placebo-controlled trial was designed to assess the efficacy of autologous bone marrow cell injection in no-option patients with severe chronic ischaemic HF. No beneficial effects of bone marrow cells were found in functional and clinical parameters or imaging modalities in patients with severe chronic ischaemic HF (compared with placebo). The study population consisted of chronic HF patients with severe left ventricular dysfunction (mean ejection fraction (EF) <35%) in NYHA score/class II–IV without any treatment options. After intramyocardial injections with autologous cell-based therapy no changes were seen with regard to EF or left ventricular volumes at 3 or 12 months. No differences were found between the cell-treated group and the placebo-treated group, the primary outcome. Regarding perfusion, at baseline stress-inducible ischaemia measured by SPECT was limited in our study population (mean summed difference score <3). No difference in myocardial perfusion, innervation or viability was measured between the cell-treated group and the placebo-treated group during follow-up. Exercise capacity and functional status remained unchanged during follow-up in both the cell-treated and the placebo-treated group. These data suggest that intramyocardial injections with autologous bone marrow cells do not improve left ventricular function or perfusion in patients with severe chronic HF. These results also imply that bone marrow cell therapy does not benefit chronic HF patients with limited stress-inducible ischaemia.

Furthermore, we aimed to investigate whether the presence of stress-inducible myocardial ischaemia influences the outcome of bone marrow cell treatment. In a prespecified subgroup analysis, only including patients with stress-inducible ischaemia, no significant changes were shown between cell-treated and placebo-treated patients in primary or secondary endpoints. However, due to the low number of patients with stress-inducible ischaemia and the small extent of stress-inducible ischaemia, this study was not accurately powered to answer this question.

Cell-based therapy has been suggested to benefit patients with ischaemic heart disease. Previously performed randomised trials demonstrated that intramyocardial bone marrow cell injection in patients with stress-inducible myocardial ischaemia and refractory angina results in improvement of cardiac function, myocardial perfusion and anginal symptoms [9–11]. Other studies with bone marrow cells could not show beneficial effects on cardiac function [25–28]. Although the majority of meta-analyses show benefit of cell-based therapy, inconsistencies in literature have been described [16, 29–31]. In a pilot study from our group, it has been suggested that bone marrow cells in patients with chronic myocardial infarction and moderate left ventricular dysfunction (mean EF 51%) lead to a decrease in HF symptoms and improved left ventricular function [13]. Other studies have shown that patients with a lower EF (<45%) after myocardial infarction benefit most from cell-based therapy [32–34]. It is not known whether this also holds true for patients with severe chronic HF (LVEF <35%). Nonetheless, the present randomised controlled trial could not confirm previous positive results. These contradictory findings might be explained by the patients’ characteristics of the different study populations.

Sustained functional improvements were described in the cell-treated group in the STAR-heart study [35]. In this study, bone marrow cells were infused via a coronary artery in patients with an EF below 35%. No data on myocardial perfusion were reported. Other randomised placebo-controlled clinical trials in chronic ischaemic heart disease patients have reported improvements in symptoms and cardiac perfusion after bone marrow cell therapy [9, 14]. However, the patient population of these latter studies had less severe left ventricular dysfunction (LVEF 40–55%) than our study population. Unlike patients with refractory angina pectoris, our study population had very limited stress-inducible ischaemia (supplementary Tab. 3) and therefore almost no complaints of angina pectoris. Due to inconsistent measuring methods of ischaemia the extent of ischaemia that is needed to profit from cell-based therapy is unidentified [11]. With the paracrine hypothesis in mind, one could imagine that the bigger the ischaemic area the more patients benefit from cell-based therapy [6, 36]. Compared with studies with a more favourable outcome, the two described dissimilarities combined in our study population, i. e. degree of left ventricular dysfunction and extent of ischaemia, may explain the results of our study. It may be that patients in our study had a too severely damaged and ‘exhausted’ myocardium to significantly increase left ventricular function and too less ischaemia to significantly improve perfusion.

Besides the results of this study, another factor needs to be addressed. As we were not able to include the intended amount of patients, the study was not sufficiently powered for our primary endpoint. Possible reasons for the inability to enrol enough patients within a realistic time frame include; exclusion of patients with other treatment options such as resynchronisation therapy or revascularisation, exclusion of patients participating in other experimental studies, and importantly also exclusion of HF patients with an EF of >35% and stress-inducible ischaemia. For eligible patients the 50% chance of randomisation to placebo was the most common reason to not participate. Since our previous randomised controlled trial showed efficacy of cell-based therapy in this population, these patients are treated in a registry [9]. The limited patient numbers might also be due to referral bias, as experimental therapies are competing with standard therapies including left ventricular assist devices in end-stage HF patients.

Because of ethical reasons to not endlessly continue a clinical trial, we decided to prematurely terminate the study four years after inclusion of the first patient, resulting in an inclusion of 39 patients. After terminating and unblinding the study, enrolment of 61% of the intended group was reached. Interestingly, there was no trend of improvement after cell-based therapy at 3 or 12 months and, what’s more, there was a trend toward a group difference in favour of placebo treatment at 12-month follow-up (*P* = 0.078). Therefore, to demonstrate the aimed improvement of 4.1% ± 5.4% difference in EF between the cell group and the placebo group, the 12 patients not included in the cell group should have improved >13% in EF compared with the 13 patients not included in the placebo group at 3‑month follow-up and >15% at 12-month follow-up. We found this an unrealistic improvement, based on previous results [9, 13, 25] and the results of this study. An interim analysis would have revealed no differences between the study groups. So, our data underline the relevance of interim analyses in clinical trials to avoid unnecessary long study duration while patients might benefit more from other treatment options.

## Limitations

Important limitations of this study with regard to the study design are the premature termination, resulting in a lack of power, as well as the fact that the study was not powered for secondary endpoint. Due to several secondary endpoints, including several time-point measurements, multiple testing was performed. Furthermore, in this prospective trial, we had to deal with missing data, and so we had to use compensatory statistics. Another limitation is that SPECT was used instead of the gold standard MRI to assess the EF, because of the high number of patients with an ICD or pacemaker.

On a more conceptual level, one could deliberate on the hypothesis that the autologous cell source in chronic HF patients may not yield the same regenerative capacity as allogeneic cells from a healthy donor. However, autologous bone marrow-derived mesenchymal stem cells in patients with severe left ventricular dysfunction did give rise to significant improvements in LVEF in the cell group vs placebo group [37]. Unfortunately, no data on stress-inducible ischaemia in this study population was reported. As other cell types, e. g. cardiac stem cells, have been suggested to be more potent during the follow-up of our study, more favourable results might have been accomplished with a different cell type. Although no differences have been described in cell retention with regard to the delivery method, the ischaemic environment may play a role in the retention and engraftment of cells after injection.

## Conclusion

In conclusion, the present study demonstrated that intramyocardial bone marrow cell injection does not improve cardiac function, or functional and clinical parameters in patients with severe chronic ischaemic heart disease and limited stress-inducible ischaemia.

### What’s new

Bone marrow cells have emerged as a potential therapy leading to angiogenesis. Here, we report a multicentre, randomised, placebo-controlled trial in which we included 39 patients with no-option chronic ischaemic heart failure and limited stress-inducible ischaemia with a follow-up of 12 months. We did not find a positive effect of cell-based therapy, not even a trend towards a beneficial effect. The fact that we did not even find the suggestion of a trend towards a positive effect in this population is a very relevant finding that adds to the growing body of evidence that mononuclear cell injection in this form is neither worth the burden for the patient nor the high costs associated with it.

## Caption Electronic Supplementary Material

Supplementary Table 1 Severe adverse events

Supplementary Table 2 Clinical and functional status

Supplementary Table 3 Effect of cell therapy on myocardial perfusion in randomised clinical trials
