# Supplementary material for: Intramyocardial bone marrow cell injection does not lead to functional improvement in patients with chronic ischaemic heart failure without considerable ischaemia
Source: Neth Heart J. 2018 Dec 19;27(2):81–92. doi: 10.1007/s12471-018-1213-2 (PMC6352621; doi:10.1007/s12471-018-1213-2)
Supplement: Supplementary file 1 — Supplementary Table 1 Severe adverse events [file 12471_2018_1213_MOESM1_ESM.docx]

Supplementary Table 1. Severe adverse events

|  | Cell group (*N*=19) | | Placebo group (*N*=20) | |
| --- | --- | --- | --- | --- |
| Dead | 2 (11%) | 1 ventricular arrhythmia  1 euthanasia | 3 (15%) | 1 acute myocardial ischaemia  1 OHCA  1 cardiorespiratory insufficiency |
| Medical intervention and exclusion from further follow-up | 2 (11%) | 2 CRT | 2 (10%) | 1 non-STEMI and CABG  1 minimal invasive MVR |
| Other | 1 (5%) | 1 sustained VT | 1 (5%) | 1 non-sustained VT |

*OHCA* out-of-hospital cardiac arrest, *CRT* cardiac resynchronisation therapy, *non-STEMI* non-ST-elevated myocardial infarction, *CABG* coronary artery bypass grafting, *MVR* mitral valve repair, *VT* ventricular tachycardia
