# Supplementary material for: Intramyocardial bone marrow cell injection does not lead to functional improvement in patients with chronic ischaemic heart failure without considerable ischaemia
Source: Neth Heart J. 2018 Dec 19;27(2):81–92. doi: 10.1007/s12471-018-1213-2 (PMC6352621; doi:10.1007/s12471-018-1213-2)
Supplement: Supplementary file 2 — Supplementary Table 2 Clinical and functional status [file 12471_2018_1213_MOESM2_ESM.docx]

| Supplementary Table 2. Clinical and functional status | | | | | | | | | | |
| --- | --- | --- | --- | --- | --- | --- | --- | --- | --- | --- |
|  | |  | **Bone marrow cell group** | | **Placebo**  **group** | | **Group difference (treatment effect)** | | | |
|  | |  | *N* | Mean+SD | *N* | Mean+SD | Estimated difference in change  (mean) | 95% confidence interval | | *P-*value |
|  | |  |  |  |  |  |  | lower | upper |  |
| NYHA | Baseline | | 19 | 2.4±.5 | 20 | 2.4±.5 |  |  |  |  |
|  | 3 mth | | 18 | 2.2±.5 | 19 | 2.3±.8 | -0.1 | -0.4 | 0.3 | 0.73 |
|  | 6 mth | | 18 | 2.3±.6 | 18 | 2.4±.6 | -0.2 | -0.5 | 0.1 | 0.13 |
|  | 12 mth | | 14 | 2.3±.7 | 14 | 2.3±.5 | -0.1 | -0.4 | 0.2 | 0.52 |
|  |  | |  |  |  |  |  |  |  |  |
| MLHF | Baseline | | 16 | 37.6±24.4 | 15 | 49.9±19.3 |  |  |  |  |
|  | 3 mth | | 14 | 29.6±21.7 | 11 | 42.0±21.4 | 0.01 | -12.9 | 13.0 | 0.99 |
|  | 6 mth | | 13 | 27.9±23.7 | 9 | 45.7±19.8 | -4.9 | -16.3 | 6.5 | 0.38 |
| Exercise capacity (W) | Baseline | | 19 | 90.8±29.2 | 19 | 84.8±32.4 |  |  |  |  |
|  | 3 mth | | 17 | 98.5±35.5 | 18 | 88.3±36.2 | 2.7 | -5.5 | 11.1 | 0.51 |
|  | 6 mth | | 17 | 95.4±35.5 | 14 | 94.0±35.5 | 4.5 | -5.4 | 14.3 | 0.36 |
| VO2 max (ml/kg/min) | Baseline | | 19 | 14.2±3.8 | 17 | 14.0±4.4 |  |  |  |  |
|  | 3 mth | | 17 | 14.5±4.2 | 16 | 13.2±3.9 | -0.2 | -2.2 | 1.7 | 0.82 |
|  | 6 mth | | 17 | 15.3±3.7 | 13 | 14.8±4.6 | 0.9 | -1.3 | 3.0 | 0.42 |

*NYHA* New York heart association score, *mth* month, *MLHF* Minnesota living with heart failure questionnaire, *VO2max* maximal oxygen consumption, *6MWT* 6-minute walk test
