# Supplementary material for: Intramyocardial bone marrow cell injection does not lead to functional improvement in patients with chronic ischaemic heart failure without considerable ischaemia
Source: Neth Heart J. 2018 Dec 19;27(2):81–92. doi: 10.1007/s12471-018-1213-2 (PMC6352621; doi:10.1007/s12471-018-1213-2)
Supplement: Supplementary file 3 — Supplementary Table 3 Effect of cell therapy on myocardial perfusion in randomised clinical trials [file 12471_2018_1213_MOESM3_ESM.docx]

Supplementary Table 3. Effect of cell therapy on myocardial perfusion in randomised clinical trials

| Author | Year | *N* cells (total) | Cell type | Baseline perfusion | Outcome |
| --- | --- | --- | --- | --- | --- |
| Losordo (33) | 2007 | 18 (24) | CD 34+ | Not reported | - |
| Ramshorst (9) | 2009 | 25 (50) | BMC | SSS 23.5, SRS 18.3 | + |
| Tse (10) | 2007 | 19 (28) | BMC | SDS 7.2 | + |
| Perin (14) | 2011 | 20 (30) | BMC | Reversible defect 18% | + |
| Perin (25) | 2012 | 10 (20) | BMC | Reversible defect 9% | - |
| Perin (34) | 2014 | 21 (27) | ADC | SDS 9.3 | + |

*BMC* bone marrow cell, *ADC* adipose-derived cells, *SSS* summed stress score, *SRS* summed rest score, *SDS* summed differences score
